# Early Clinical Outcomes and Advantages of a Novel-Size Adjustable Second-Generation Cryoballoon: A Proof-of-Concept Study

**DOI:** 10.3390/jcm13051259

**Published:** 2024-02-22

**Authors:** Marco Schiavone, Gaetano Fassini, Massimo Moltrasio, Benedetta Majocchi, Fabrizio Tundo, Francesca Casati, Claudio Tondo

**Affiliations:** 1 Department of Clinical Electrophysiology & Cardiac Pacing, Centro Cardiologico Monzino IRCCS, 20138 Milan, Italy; 2 Department of Systems Medicine, University of Rome Tor Vergata, 00133 Rome, Italy; 3 Boston Scientific Italy, 20134 Milan, Italy; 4 Department of Biomedical, Surgical and Dental Sciences, University of Milan, 20100 Milan, Italy

**Keywords:** atrial fibrillation, catheter ablation, cryoballoon, adjustable, POLARx FIT

## Abstract

**(1) Background/Objective** Balloon-guided catheter ablation (CA) has emerged as an alternative option for atrial fibrillation (AF) management. The recent introduction of a novel-size adjustable second-generation cryoballoon (CB) system offers innovations, but clinical outcomes remain unexplored. This study aims to assess the acute performance of the POLARx FIT™ CB system in AFCA. **(2) Methods:** Consecutive patients undergoing AF ablation with the POLARx FIT™ CB system in our center were included. The primary outcome was the rate of 31 mm balloon-size utilization, with secondary outcomes including acute pulmonary vein isolation (PVI) rate, periprocedural complications, and in-hospital AF recurrences. **(3) Results:** Twenty-four patients with a mean age of 59.5 years, predominantly male (87.5%), and exhibiting paroxysmal AF (91.7%) were enrolled. Procedural characteristics demonstrated a high acute success rate (100% PV isolation) with a favorable safety profile. Notably, the 31 mm CB configuration was utilized in 51% of applications, showcasing its adaptability in challenging anatomies. No major complications occurred, with two patients experiencing in-hospital self-limiting AF recurrences. **(4) Conclusions:** This study represents the first comprehensive assessment of the POLARx FIT™ CB system in AF ablation. While acknowledging the study’s limitations, this novel CB emerges as a promising tool, warranting further exploration in larger studies with extended follow-up periods.

## 1. Introduction

Balloon-guided catheter ablation (CA) has established itself as a prominent treatment option for atrial fibrillation (AF) management [1,2]. In comparison to traditional radiofrequency (RF) ablation, cryoballoon (CB) ablations have proven to achieve durable and effective pulmonary vein isolation (PVI) in various trials [3,4]. These systems offer potential advantages over traditional RF procedures, addressing issues such as the extended learning curve required for RF procedures and the need for sufficient dexterity to create precise contiguous circular lesions. Most trials have focused on the evaluation of the 23 or 28 mm Arctic Front Advance™ CB (Medtronic, Inc., Minneapolis, MN, USA), progressing through different generations, including CB-2, CB-3, and CB-4 (second-, third-, and fourth-generation balloons). These iterations featured improved distal hemisphere freezing, a shorter distal nose-tip for the real-time assessment of PV potentials, and enhanced catheter maneuverability, leading to shorter procedural times, while maintaining comparable safety and improved efficacy [5,6].

In recent years, a competing CB system, POLARx™ (Boston Scientific, Marlborough, MA, USA), with a similar workflow and some technical enhancements, has gained global usage. Initial comparisons indicate similar acute outcomes between the two systems, with the POLARx system demonstrating a higher rate of time-to-isolation (TTI) recording in the inferior PVs and achieving lower nadir temperatures [7,8,9]. The POLARx system has also evolved with the introduction of its second generation, known as POLARx FIT™. This novel CB features a larger surface area, enhancing its ability to conform to variable anatomies and expanding therapeutic possibilities. Additionally, it is the only CB with a dual-diameter balloon size in one catheter, offering elasticity to accommodate surfaces ranging from 28 mm to 31 mm. This provides a 20% increase in surface area for occluding and treating variable anatomies, minimizing the need for repositioning, and enabling more antral placement [10]. Despite technical advancements, there are currently no available studies in the literature on the clinical outcomes of AF catheter ablation using the POLARx FIT™ system. Herein, we present our early results with the POLARx FIT™ second-generation CB system.

## 2. Methods

This study constituted a single-arm observational investigation assessing the short-term outcomes of patients undergoing AF ablation utilizing the POLARx FIT™ CB system. The study complied with the Declaration of Helsinki and its subsequent amendments. Written informed consent for the procedures was obtained from all participating patients.

### 2.1. Study Population

All consecutive patients who underwent catheter ablation for AF with the POLARx FIT™ CB system at our institution, Centro Cardiologico Monzino in Milan, Italy, between May 2023 and November 2023, were included in this study. Choice between ablation techniques available in our center was left to operator discretion. Therefore, only patients undergoing POLARx FIT™ CA according to operator choices were included in this observational project. Choices were mostly made according to ablation subtype (paroxysmal rather than persistent) as well as clinical characteristics. Exclusion criteria for CA encompassed individuals below the age of 18, severe left atrium (LA) dilation (LA diameter exceeding 55 mm), the presence of intracardiac LA thrombi, acute heart failure, severe valvular heart disease, and contraindications to oral anticoagulation (OAC).

### 2.2. Ablation Procedures

Patients in AF prior to the procedure underwent preprocedural transesophageal echocardiogram to rule out intracardiac thrombi. Continuous anticoagulation with direct anticoagulants was implemented, and for those receiving vitamin K antagonists, the targeted international normalized ratio ranged between 2 and 3. All procedures were conducted under conscious or deep sedation utilizing a combination of midazolam, propofol, fentanyl, and/or dexmedetomidine. Esophageal temperature during CB ablation procedures was monitored using an esophageal probe. The cut-off temperature for CB ablation was established at 18 °C. In instances where the esophageal temperature fell below this cut-off value, energy delivery was terminated prematurely. To assess phrenic nerve capture during ablation of the right superior and inferior pulmonary veins (RSPVs, RIPVs), either a decapolar or a quadripolar catheter was positioned in the superior vena cava. Following transseptal puncture, heparin boluses were administered to maintain an activated clotting time of ≥300 s throughout the entire procedure.

The general characteristics of POLARx FIT CB ablation did not exhibit significant differences from the preceding generation of the POLARx CB procedure, which has been previously elucidated [8]. Following LA access achieved through a single transseptal puncture, the POLARx FIT CB was introduced into the LA under the guidance of a spiral catheter (POLARMAP™ Circular Mapping Catheter). This was facilitated through its dedicated steerable sheath (POLARSHEATH™) after exchanging the over-the-wire sheath with the SL0 or SL1 sheath utilized for the transeptal puncture, as previously described. The spiral catheter was optimally positioned to record PV potentials. The CB was then positioned at the antrum of a PV and advanced toward the PV. Fluoroscopy and contrast medium injection through the balloon’s distal tip were used to assess local PV occlusion. The freezing time duration and number of attempts for each vein, as well as any additional lesions or procedures, were left to the operator’s discretion, typically with application durations ranging from 180 to 240 s. If isolation information regarding the time was unavailable, the CB was retracted after freezing to the angiographically defined PV ostium, and PVI was reevaluated. If PVI was unsuccessful, supplementary bonus freezes were administered, with no waiting time applied. The choice between the 28mm or 31mm size was made by the operator based on PV anatomy and occlusion characteristics. Following ablation, PVI was evaluated by confirming entrance block through the absence of vein potentials, and exit block was verified by the failure to capture the left atrium (LA) upon stimulation inside the vein with maximum output. The presence of pericardial effusion was assessed either by transthoracic or intracardiac echocardiography at the conclusion of the procedure.

### 2.3. Data Collection and Outcome Definition

Demographic information, personal medical history, AF characteristics, cardiovascular risk factors, and peri-procedural data were systematically documented for all patients in a centralized de-identified database. The primary focus of the study was to determine the rate of 31 mm balloon-size utilization. The assessment of procedural CB performance, specifically in terms of acute PVI rate and periprocedural complications, was considered a secondary outcome.

### 2.4. Statistical Analysis

Normality of the distribution of all continuous variables was tested with the Shapiro–Wilk test. Continuous variables were reported as mean ± standard deviation (s.d.) or as median [inter-quartile range (1st–3rd quartile)) if normally or non-normally distributed, respectively. Categorical variables were reported as count (%). Data analysis was performed using STATA 14.0 (StataCorp LLC, College Station, TX, USA).

## 3. Results

### 3.1. Patient Population

A total of 24 patients undergoing AF ablation with the POLARx FIT CB were included in the study. The mean age of the cohort was 59.5 ± 13.0 years, and 87.5% were males. Median body mass index was 24.3 ± 2.5 kg/m^2^. The AF pattern was paroxysmal in 91.7% of cases. Hypertension was the most common comorbidity in the cohort (29.2%), while dyslipidemia was present in 29.2% of patients, and chronic kidney disease was shown in 4.2%. No heart failure (HF) patients were enrolled in this project, while three patients were valvular heart disease patients (one was treated with a previous valvular heart replacement) and two were chronic coronary syndrome patients, previously treated with a percutaneous coronary intervention (PCI). Median left atrial (LA) dimensions were overall normal, with a median LA diameter of 36.0 mm [IQR 30.0–41.0) and median LA volume of 68.0 mL (51.0–79.0). Median left ventricular (LV) function was overall normal, with a median LV ejection fraction (LVEF) of 61.5% (IQR 57.0–66.2%). On admission, 75.0% of patients were on oral anticoagulant therapy (OAC) and 52.8% were on antiarrhythmic drug (AAD) therapy, mostly on IC AADs; 25% of patients were on beta-blockers. The overall characteristics of the study cohorts are reported in Table 1.

### 3.2. Procedural Characteristics

Eighteen patients (75.0%) were in sinus rhythm at the beginning of the procedure. All procedures were performed using the POLARx FIT CB. No additional RF touches were needed to obtain PVI since 100% of the PVs were isolated by means of the CB at the end of the procedures. A total of 124 CB applications were needed to isolate all PVs, 64 (51.6%) using the 31 mm size. In 16/24 cases (66.6%), at least 1/4 PVs required the use of 31 mm CB size. Median procedural time and fluoroscopy time were 90.0 (IQR 60.0–110.0) mins and 15.5 (IQR 12.0–22.3) mins, respectively, with a median LA dwell time of 44.5 (IQR 36.5–50.5). All procedures were performed using esophageal temperature monitoring. One patient presented a left common pulmonary trunk and one patient presented both a left and a right common pulmonary trunk; all other patients presented a separate origin of both left and right PVs. Three patients presented an additional PV branch, with an independent origin. Minimal reached temperatures were as follows: LSPV −52.0 (IQR −54.5–−50.5) °C; LIPV −49.0 (IQR −52.0–−48.0) °C; RSPV −50.0 (IQR −54.0–−45.5) °C; RIPV −50.0 (IQR −57.0–−48.0) °C. In 12.5% of cases, additional RF ablations were performed to isolate the cavo-tricuspid isthmus due to a clinical history of atrial flutter.

No major periprocedural complications were observed in the study cohort; two patients experienced AF recurrences (8.3%), self-limiting during the admission. One mild groin hematoma was detected. All patients were discharged in sinus rhythm, both with antiarrhythmic (16.6% with amiodarone, the others with flecainide or propafenone) and anticoagulation therapy. A complete list of peri-procedural characteristics is reported in Table 2.

## 4. Discussion

To the best of our knowledge, this study represents the first full report assessing the performance of the POLARx FIT CB. The main results of our study are as follows:

- In an unselected cohort of patients undergoing CB ablation with the POLARx FIT, the 31 mm size CB configuration was used for 51% of the overall applications.
- In several clinical scenarios, the use of this new CB feature has helped the operator to reach an easier PVI, such as in cases with common PV trunks, accessory PVs, and funnel-shaped PV ostia.
- The new POLARx FIT confirmed the easy maneuverability of the previous POLARx CB, with an overall high acute success rate and low periprocedural complication rate/in-hospital AF recurrences.

### 4.1. Procedural Aspects

Balloon-based CA techniques hold significant potential for simplifying the complexity associated with 3D mapping system-guided RFCA. These techniques offer the promise of reducing overall procedural time while maintaining comparable safety and efficacy profiles, particularly in patients with paroxysmal AF [3,11]. While the AFA-Pro CB has been extensively documented in the literature, particularly for its efficacy and safety in paroxysmal AF catheter ablation [6,12,13], data on the newer PolarX are relatively scarce, especially concerning mid-term efficacy and safety [7,14]. Despite these differences, the PolarX balloon offers distinct advantages, notably its ability to maintain consistent inner pressure during freezing. Initially hailed for its potential to enhance PVI and minimize the pop-out phenomenon, thereby improving contact with the tissue, comparative studies have shown no significant differences in the total number of freeze applications and applications per pulmonary vein between the two technologies. In comparison to previous analyses, our study found similar procedural times to Tanese et al. [7] and lower times than Guckel et al. [14]. This suggests that while the PolarX FIT may not significantly reduce procedural or fluoroscopy times, especially in cases of standard pulmonary vein anatomy, its main advantage lies elsewhere. It is worth noting that the presence of PV common trunks was minimal in our cohort, indicating minimal influence on overall procedural times. While CB procedures generally achieve faster PVI compared to radiofrequency catheter ablation due to their single-shot nature, certain clinical scenarios can still present challenges for traditional CBs due to their fixed sizes. Studies have shown significantly shorter procedure and ablation times in groups using adjustable-size CBs [15,16,17,18], such as those reported by Shi et al. [19] and Hoffmann et al. [20], compared to radiofrequency catheter ablation. This difference may also be attributed to study protocols involving three-dimensional mapping before and after PVI, contributing to longer procedure durations, particularly in cryoballoon ablation where mapping is less routine. While anatomical variations can pose challenges and prolong CB procedures, performing computed tomography scans before procedures to guide balloon selection based on anatomy may not be feasible in most centers during routine clinical practice [19]. Therefore, the availability of adjustable-size CBs offers significant flexibility to electrophysiologists.

Before the introduction of POLARx FIT to the market, the availability of only two CB sizes (23 mm and 28 mm) limited the adaptability of CB procedures, particularly in cases involving anatomical variants such as common trunks, accessory veins, and large PV ostia. These variations often impact the procedural workflow, necessitating multiple CB repositioning, segmental approaches, and distal CB positioning to achieve adequate PV isolation, potentially leading to peri-procedural complications such as phrenic nerve palsy (PNP) and bronchial hemorrhage [21].

The association between anatomy and the safety or efficacy of CB ablation has recently been emphasized in a meta-analysis by Hayashi et al. [22]. Various anatomic characteristics have been identified as predictors of acute success, including a left lateral ridge <4.7 mm for the left superior PV (LSPV) and a PV ostium-to-bifurcation distance ≤10.4 mm for the right inferior PV (RIPV). Larger right superior PV (RSPV) ostia have also been linked to post-CBCA recurrences, as reported by Güler et al. [23].

### 4.2. Case-Based Approach

**Figure 1:**
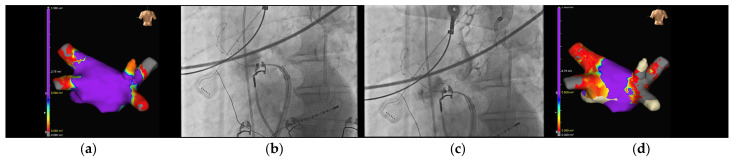
CB ablation of an angled posterior and vertical branch of the RSPV, originating from a large-shaped PV ostium. From left to right: (**a**) 3D mapping showing RSPV anatomy. (**b**) Unsuccessful attempt to occlude and isolate this branch with the 28 mm size CB. (**c**) Successful attempt to occlude and isolate this branch with the 31 mm size CB. (**d**) Three-dimensional mapping showing RSPV after cryoenergy delivery.

**Figure 2:**
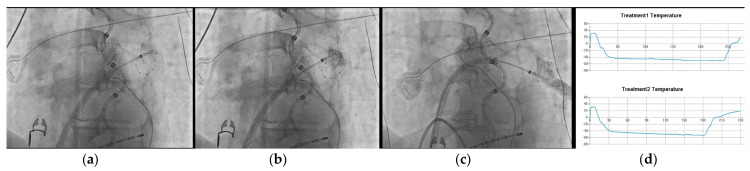
CB ablation of a left PV common trunk. (**a**) Unsuccessful attempt to occlude the common trunk with the 28 mm size. In this case, the 28 mm size was used to further advance the CB towards the vein. The CB was subsequently pulled back towards the antrum while changing its size from 28 to 31 mm. (**b**,**c**) Double-shot energy delivery with the 31 mm CB to isolate the LPV common trunk. (**d**) Profile temperature recorded by the POLARX FIT remote console.

**Figure 3:**
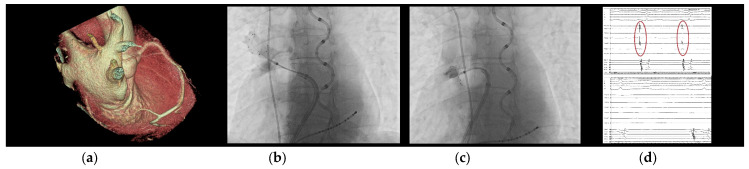
CB ablation of an additional branch of the RSPV, with a complex take-off angle. From left to right: (**a**) CT imaging of this additional RSPV branch, vertically oriented. (**b**) Unsuccessful attempt to occlude and isolate this branch with the 28 mm size CB. (**c**) Successful attempt to occlude and isolate this branch with the 31 mm size CB. (**d**) POLAR map recorded electrograms in this additional RPV branch, pre- and post-cryoenergy delivery.

When comparing the previous generation of this balloon with this second one, the PolarX FIT has a dual-diameter balloon size, offering elasticity to accommodate surfaces ranging from 28 mm to 31 mm. This intraprocedural flexibility afforded by the availability of different balloon sizes resulted in the predominant use of the larger variant in most freeze applications. Notably, in 66.6% of cases, both balloon sizes were employed to ensure optimal occlusions for all veins. The introduction of this second-generation CB with its 31mm configuration was indeed particularly noteworthy for our cases’ management as it enhanced the quality of occlusion. This innovation reduced the necessity for CB repositioning and segmental approaches for PCTs and veins with multiple branches in our cohort, which are potential predictors of worse outcomes [24], as previously mentioned. Consequently, the POLARx FIT™ is likely to increase the rate of single-shot isolations in the future. Manuscript’s figures provide a visual summary of the advantages of this novel CB in three challenging scenarios for a traditional CB: angled branches originating from a large ostium (Figure 1), PCTs (Figure 2), and additional branches (Figure 3).

The increased antral occlusion facilitated by the 31 mm size potentially reduces the risk of phrenic nerve injury, with none reported in our cohort. The characteristics of this novel-size adjustable CB may mirror the advantages of the laser balloon (LB), allowing variable sizing and deformation for adaptability to the highly variable sizes and shapes characteristic of PVs [25,26]. This adaptability aids in sensing electrograms and ascertaining TTI. The variable and adapting size of the CB assists in creating more antral lesions for improved substrate modification, akin to the LB. This is of pivotal importance as PVI performed with a wide antral approach has been shown to be more effective than ostial PVI in achieving freedom from arrhythmia recurrences at long-term follow-up [27]. This approach is particularly effective in eliminating reentry wavelets localized around the PV antrum. In addition to more extensive debulking of the LA and CA of non-PV foci in the posterior LA wall, antrum PV isolation may successfully ablate ganglionic plexi, particularly those located at the border of the PVs [28]. On the other hand, it is important to acknowledge that wider lesions might potentially pose a risk of arrhythmic isthmus formation in the posterior wall or the mitral isthmus. However, given the slight increase in balloon surface area, this risk appears to be negligible.

### 4.3. Limitations

The main constraints of our study stem from the limited size of our participant pool and the absence of extended-term efficacy data for this innovative CB, which hinder our ability to make conclusive statements regarding its effectiveness. However, it is essential to underscore that our analysis stands as the initial comprehensive examination of this technology in the literature. Another secondary limitation to acknowledge is the single-arm design of the study, which restricts our capacity to compare outcomes with alternative treatments. Nonetheless, the unique workflow implemented with this device, being the sole adjustable-size CB, precludes extensive direct procedural comparisons with other devices lacking similar attributes.

## 5. Conclusions

This study represents the first comprehensive assessment of the POLARx FIT™ CB system in AF ablation. The POLARx FIT™ distinguishes itself as the sole CB offering two balloon sizes within a single catheter, presenting the potential to simplify the treatment of complex anatomies and harness all the benefits associated with increased antral occlusion. In a single-center cohort, 33 mm was used in most cases. While acknowledging the study’s limitations, the POLARx FIT™ emerges as a promising tool, warranting further exploration in larger studies with extended follow-up periods.

## Figures and Tables

**Table 1:** Baseline characteristics of the study cohort.

	Overall (*n* = 24)
Age, mean ± s.d.	59.5 ± 13.0
Male sex, *n* (%)	21 (87.5)
AF Type	
Paroxysmal AF, n (%)	22 (91.7)
Persistent AF, n (%)	2 (8.3)
AF duration (months), median (IQR)	23 (8–84)
BMI, mean ± s.d.	24.3 ± 2.5
Hypertension, *n* (%)	7 (29.2)
Dyslipidemia, *n* (%)	4 (16.6)
CAD, *n* (%)	2 (8.3)
Valvular heart disease, *n* (%)	3 (12.5)
CKD, *n* (%)	1 (4.2)
Palpitations, *n* (%)	21 (87.5)
EHRA Score Class	
1, *n* (%)	3 (12.5)
2a, *n* (%)	15 (62.5)
2b, *n* (%)	5 (20.8)
3, *n* (%)	1 (4.2)
**LA volume index (mL/m^2^), median (IQR)**	35.5 (25.4–39.0)
LVEF, mean ± s.d.	61.0 (57–66)
Drug therapy at baseline	
OACs, *n* (%)	18 (75.0)
VKAs, n (%)	0 (0)
Direct OACs, n (%)	18 (75.0)
AADs, *n* (%)	58 (52.8)
IC, *n* (%)	18 (75.0)
Amiodarone, *n* (%)	1 (4.2)
Sotalol, *n* (%)	0 (0)
Beta-blockers, *n* (%)	6 (25.0)

Abbreviations: AADs = antiarrhythmic drugs; AF = atrial fibrillation; BMI = body mass index; CAD = coronary artery disease; CKD = chronic kidney disease; IQR = interquartile range; LA = left atrium; LVEF = left ventricular ejection fraction; OACs = oral anticoagulants; s.d. = standard deviation; VKAs = vitamin K antagonists.

**Table 2:** Periprocedural characteristics and study outcomes.

	Overall (*n* = 24)
Sinus rhythm at index procedure, *n* (%)	18 (75.0)
Overall procedural time (min), median (IQR)	90 (60–110)
Overall fluoroscopy time (min), median (IQR)	15.5 (12–22.3)
Use of esophageal temperature probe, *n* (%)	24 (100)
Anatomical variants	
Right common pulmonary trunks, *n* (%)	2 (8.3)
Left common pulmonary trunks, *n* (%)	1 (4.2)
Additional PV branches, *n* (%)	3 (12.5)
Number of applications needing the 31 mm size, *n* (%)	64 (51.6)
Minimal reached temperatures	
LSPV (°C), median (IQR)	−52.0 (−54.5–−50.5)
LIPV (°C), median (IQR)	−49.0 (−52.0–−48.0)
RSPV (°C), median (IQR)	−50.0 (−54.0–−45.5)
RIPV (°C), median (IQR)	−50.0 (−57.0–−48.0)
In-hospital AF recurrences, *n* (%)	2 (8.3)
Periprocedural complications	
Groin hematoma, *n* (%)	1 (4.2)
Pericardial effusion, *n* (%)	0 (0)
Cardiac tamponade, *n* (%)	0 (0)
Phrenic nerve palsy, *n* (%)	0 (0)
Thromboembolic complications, *n* (%)	0 (0)
Drug therapy at discharge	
OACs, *n* (%)	24 (100)
VKAs, *n* (%)	0 (0)
Direct OACs, *n* (%)	24 (100)
AADs, *n* (%)	58 (52.8)
IC, *n* (%)	20 (83.3)
Amiodarone, *n* (%)	4 (16.7)
Sotalol, *n* (%)	0 (0)
Beta-blockers, *n* (%)	6 (25.0)

Abbreviations: AADs = antiarrhythmic drugs; AF = atrial fibrillation; IQR = interquartile range; LSPV = left superior pulmonary vein; LIPV = left inferior pulmonary vein; OACs = oral anticoagulants; RSPV = right superior pulmonary vein; RIPV= right inferior pulmonary vein; VKAs = vitamin K antagonists.

## Data Availability

The data that support the findings of this study are available on reasonable request from the corresponding author.
